# The Masquelet technique in traumatic loss of the talus after open lateral subtalar dislocation—A case report

**DOI:** 10.1016/j.ijscr.2019.10.029

**Published:** 2019-10-21

**Authors:** Ahmed Nabil Abdulazim, Martina Reitmaier, Henrik Eckardt, Rik Osinga, Franziska Saxer

**Affiliations:** a Department of Orthopaedic and Trauma Surgery, University Hospital Basel, Spitalstrasse 21, 4031, Basel, Switzerland; b Department of Plastic, Reconstructive, Aesthetic and Hand Surgery, University Hospital of Basel, Spitalstrasse 21, 4031, Basel, Switzerland; c Crossklinik, Swiss Olympic Medical Center, Bundesstr. 1, 4054, Basel, Switzerland

**Keywords:** Case report, Traumatic loss talus, Masquelet technique, Hindfoot arthrodesis

## Abstract

**Introduction:**

Subtalar dislocations are rare injuries and treatment recommendations missing. Gross contamination and devascularisation are aspects supporting talectomy as potential treatment choice. Reconstruction in these cases can be challenging. The Masquelet technique presents one viable option.

**Presentation of case:**

A carpenter sustained a high energy chrush injury with traumatic open lateral talar dislocation qualifying as Gustillo Anderson type IIIc injury, a mangeled extremity index of 7 and gross contamination. The severity of soft tissue damage and contamination prompted the decision for talectomy before plastic coverage with a gracilis flap. Additionally a Masquelet procedure with cement spacer was initiated.

After consolidation of the soft tissues a hindfoot arthrodesis with approximate preservation of leg length could be performed.

In the course of treatment, the patient suffered no infection and could resume full weight bearing after nine months. The patient resumed his previous occupation with adapted workload and is satisfied with the treatment result.

**Discussion:**

In the present case the principle options were talus preservation or talectomy with reconstruction. Talus preservation in the presence of gross contamination can be associated with the risk of infection, in addition there is a risk to develop secondary arthritis. For this individual patient the a fast and definite solution was important at this point in life. Surgeons therefore opted for talectomy. To optimally reconstruct leg length and optimize for arthrodesis the Masquelet technique was employed.

**Conclusion:**

The Masquelet technique is a viable option also in the emergency situation to preserve extremity length and optimize tissue viability.

## Highlights

• The Masquelet technique is suitable for emergency treatment and later reconstruction.
• The Masquelet technique is an option even in presence of contamination and vascular impairment.
• In case of traumatic bone loss, the Masquelet technique can preserve leg length not only in long-bone defects.
• For grafting procedures the Masquelet technique can improve the viability at the recipient site.
• The biologic activity of the Masquelet membrane facilitate union at challenging graft-sites.

## 1 Introduction

Subtalar dislocation is a very rare injury representing only 1 % of all traumatic dislocations [1]. The injury can be associated with a varying degree of soft tissue damage from closed to Gustillo Anderson IIIC lesions [2]. The dislocation is typically classified according to the direction of dislocation (medial, lateral, anterior and posterior). Lateral subtalar dislocations (in 26 % of the cases) are reportedly associated with a worse outcome than medial ones [3]. About 22.5 % of all subtalar dislocations are open and the majority of these cases is accompanied by a fracture (61.4 %) [4]. In general open lateral subtalar dislocation has the worse outcome among all types of subtalar dislocations with poor results in 81.8 % [4]. Gross contamination of the talus or disruption of the blood supply possibly associated with open dislocation can prompt the decision for primary talectomy [5]. There is currently no consensus on the optimal treatment of traumatic loss of the talus. Here, we present the case of 45-year-old man with primary talectomy after open lateral subtalar dislocation and augmentation of the defect before arthrodesis using the Masquelet technique [6].

This work has been reported in line with the SCARE criteria [7], the checklist is available as supplemental material.

## 2 Case

**Fig. 1:**
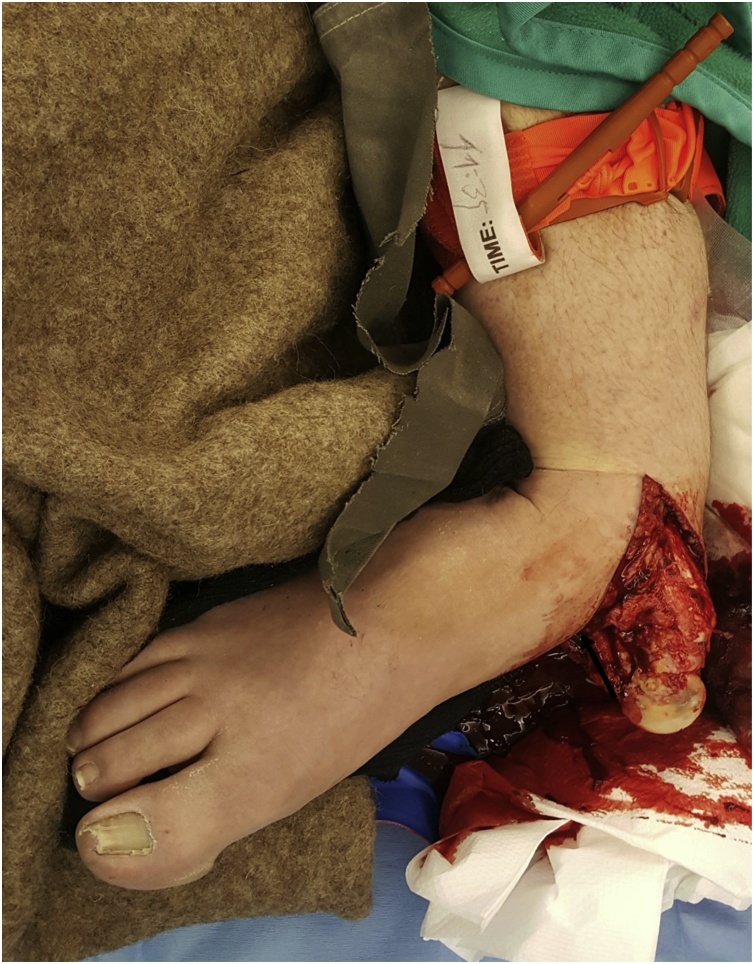
Initial presentation with externalised talar head (arrow).

We present the case of a 45-year-old heavy-built independent master carpenter who was buried under several wooden boards with a total weight of 800 kg. The patients past medical history was characterized by the diagnosis of narcolepsy some years previous, stabilization of a fracture at the L2 level, sleep apnea with a body mass index of 35 kg/m^2^ and arterial hypertension. The patient however was not under any long-term medication. He was brought to our emergency room of a level 1 trauma centre via emergency medical services. The initial examination revealed an open lateral subtalar dislocation of the right foot with the talus and distal tibia completely exposed and contaminated with small pieces of wood and sawdust (Fig. 1). The patient reported intact sensation of the whole foot. He had palpable dorsalis pedis but no posterior tibial pulses. Because of heavy bleeding from the wound a tourniquet had been applied.

**Fig. 2:**
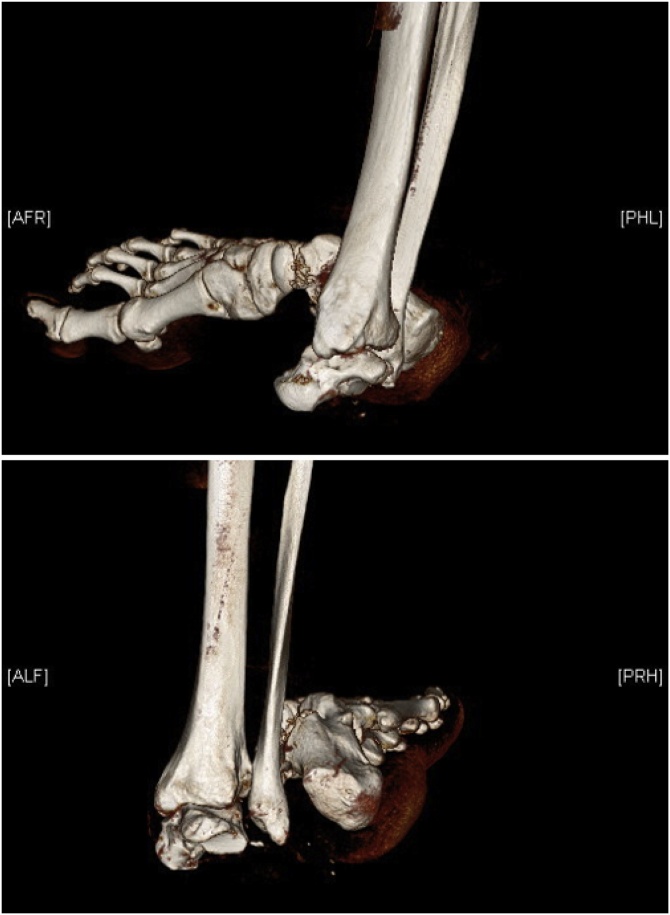
Initial presentation on computer tomography.

Computed tomography further revealed a pelvic fracture that did not require surgical intervention. There was no major fracture of the talus, only small avulsions in the subtalar joint (Fig. 2).

A tetanus vaccination was administered and antibiotic treatment with amoxicillin/clavulanic acid established. With the diagnosis of an IIIC open lateral subtalar dislocation the patient was transferred to the operating room 70 min after the accident with a total tourniquet time of 40 min. The patient had a MESS (mangeled extremity severity score) of 7 (see Table 1, transient hypotonia in view of an initial loss of consciousness most likely as part of a situational syncope).

**Table 1:** Mangled Extremity Severity Score.

Tissue Injury	Description	Score Points
Low energy	stab wound, simple fracture, low energy gunshot wound	1
Medium energy	open or multiple fractures, dislocation	2
High energy	high speed motor vehicle collision or rifle gunshot wound	3
Massive crush	above plus gross contamination	4
Shock		
Normotension	Systolic blood pressure always >90 mmHg	0
Transient hypotension	Systolic blood pressure transiently <90 mmHg	1
Hypotension	Systolic blood pressure persistently <90 mmHg	2
Ischemia^a^		
None		0
Mild	Pulse reduced or absent but perfusion normal	1
Moderate	Pulseless; paresthesia, diminished capillary refill	2
Advanced	Cool, paralyzed, insensate, numb	3
Age		
<30 yrs		0
30–50 yrs		1
>50 yrs		2

a Double in case of ischemia >6 h.

**Fig. 3:**
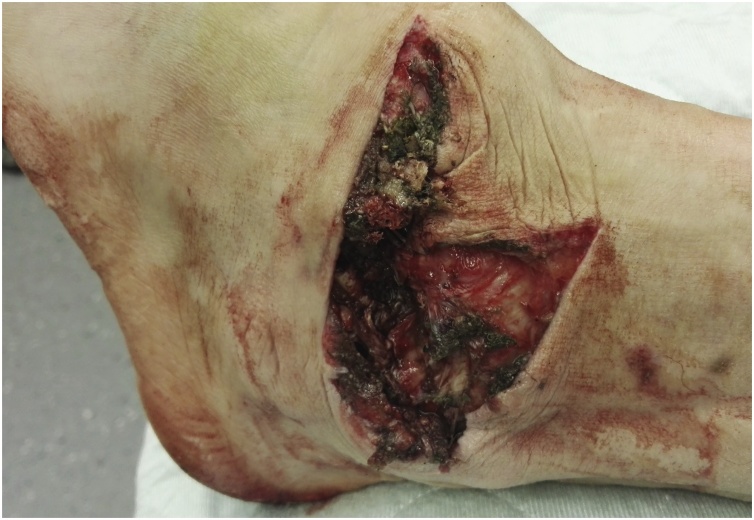
Clinical picture after reduction.

**Fig. 4:**
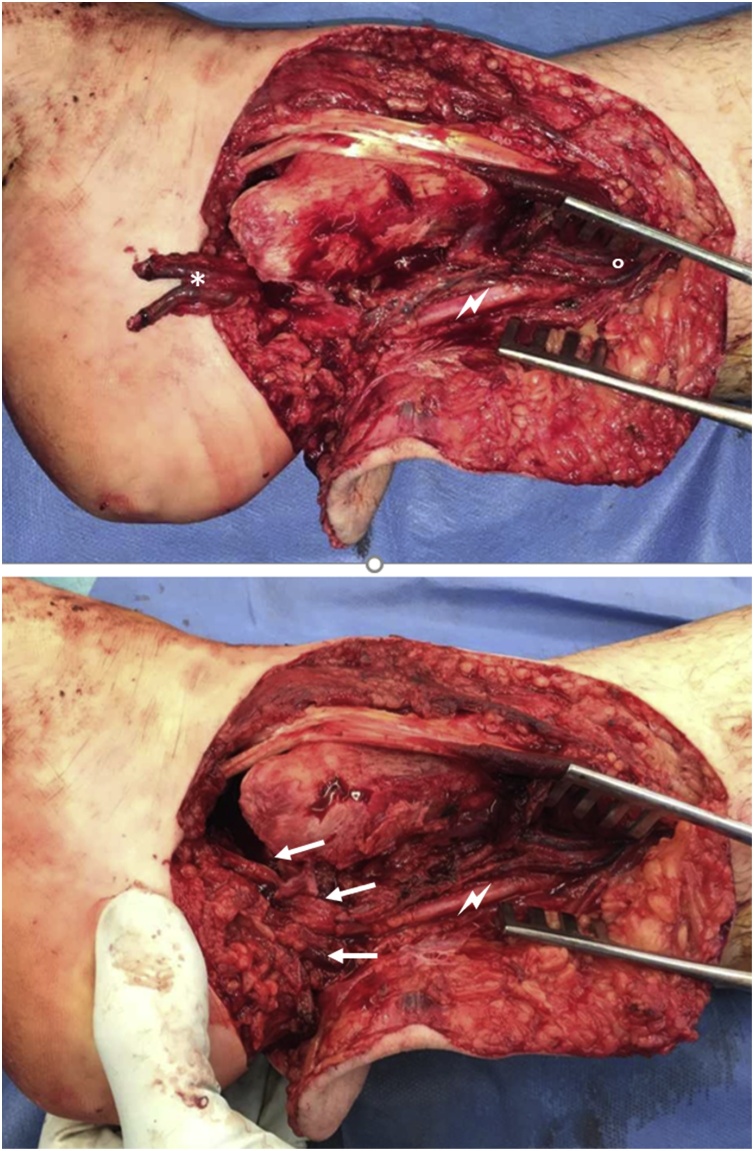
Intraoperative situs, the distal vascular bundle is marked *, the proximal one °, note the extensive contusions of the vessels and the consequent segmental loss of vasculature. The nerve is marked  with the trifurcation distally shown using arrows.

The subtalar joint was reduced and the wound was thoroughly irrigated. The subtalar joint had lost a significant amount of its cartilage. The medial soft tissue envelope was avulsed from the medial malleolus and talus and also the posterior structures were detached from the posterior tibia and talus, while the anterolateral structures were intact. The dorsal tibial artery was disrupted with macroscopic adventitial damage of 5 cm length both of the proximal and distal stump. The tibial nerve was in continuity trifurcating just below the wound, which explains the intact sensation of the sole of the foot (see video included in the supplementary material and Fig. 3, Fig. 4). No bleeding could be observed after drilling of the talus.

We opted for primary talectomy and decided to employ the Masquelet technique for reconstructing the defect.

**Fig. 5:**
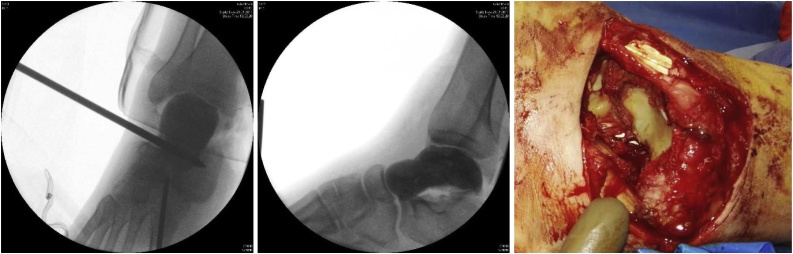
Cement spacer in fluoroskopic view and during surgery.

In preparation for the defect augmentation using the Masquelet technique the joint was debrided, the remaining cartilage in the talar joints was removed and a talus shaped cement spacer was implanted (Fig. 5). The patient had an external fixator mounted and the soft tissue defect was temporarily closed using a vacuum dressing.

**Fig. 6:**
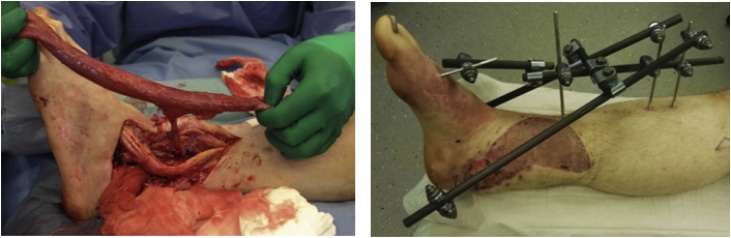
Free gracilis flap before anastomosis proximal to the injured zone and postoperative result after split skin graft and external fixation.

Three days later the vacuum dressing was changed and the spacer renewed with further debridement, tissue biopsies for microbial analysis and irrigation of the wound. Seven days after primary surgery the soft tissue defect was reconstructed using a contralateral free gracilis muscle flap covered with split-thickness skin graft from the ipsilateral thigh, anastomosed proximal to the zone of injury in an end-to-end fashion to the dorsal tibial artery and two concomitant veins. The wound healed well with no signs of infection. The patient was discharged three weeks after the accident with the external fixator and non-weight bearing mobilization (Fig. 6).

**Fig. 7:**
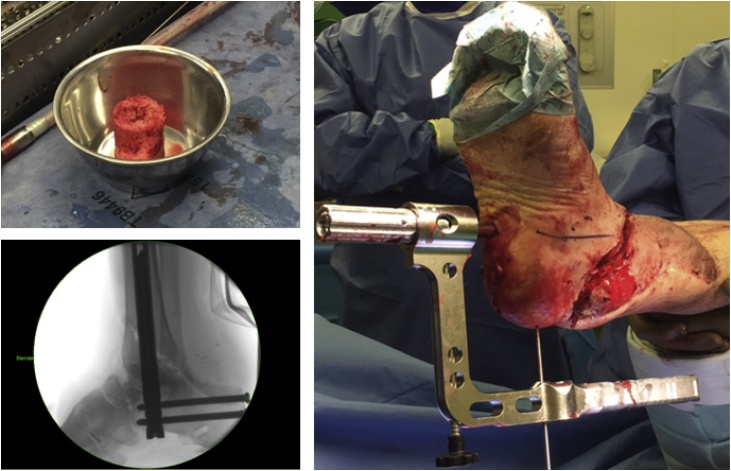
Harvested bone graft before implantation, fluoroscopic view of arthrodesis and graft, intraoperative view.

At six weeks after primary surgery, the patient was prepared for the next stage using the Masquelet technique. The external fixator was removed and the gracilis flap was elevated. The membrane that had formed around the cement spacer was carefully opened and the spacer removed. A RIA (reamer irrigator aspirator) system was used to harvest bone graft to fill the defect within the membrane. Finally, a 240 mm × 12 mm arthrodesis nail was implanted for fusion of the hindfoot (Fig. 7).

**Fig. 8:**
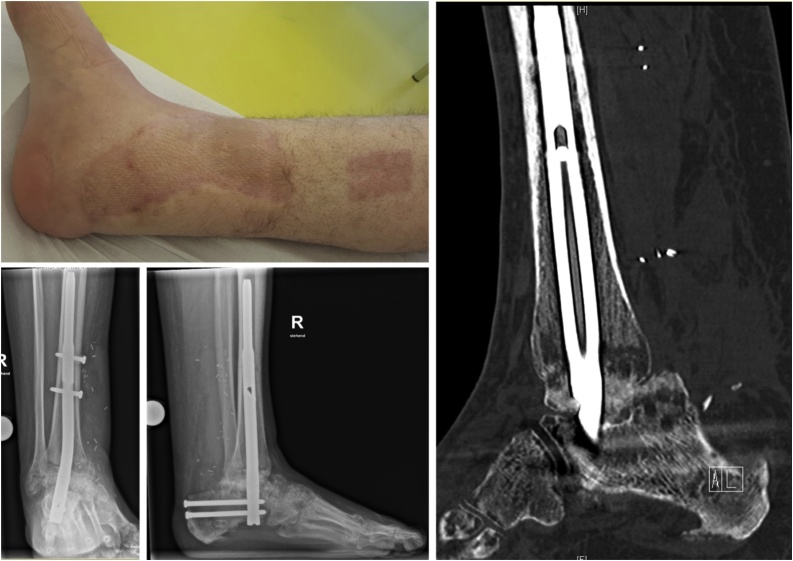
Results after 9 months with full soft tissue coverage and beginning osseous consolidation.

The patient remained mobilized with partial weight bearing with a maximum of 10 kg for the next six months. The wound healed uneventfully. Radiographs were repeatedly taken to assess osseous fusion. After nine months a CT scan showed progressive osseous fusion and full weight bearing was allowed (Fig. 8). The At twelve months after injury, the patient was walking with a slight limp and only little pain at rest. He was able to return to his work as a carpenter with adapted footwear and workload (i.e. avoidance of uneven underground, long working days and heavy loads). The leg length difference war 1 cm and did not subjectively affect the patient.

## 3 Discussion

Open peritalar dislocation is a rare but devastating injury. It can lead to deep infection, osteonecrosis, posttraumatic osteoarthritis and in cases of total loss of the talus to leg length discrepancy [8].

For many years the Masquelet technique has been successfully used in the reconstruction of osseous defects of the long bones [9]. In a first step, a spacer is implanted in the defect site. This allows restoration of the length and also helps control the infection. In cases of severe soft tissue injury, the use of a flap can be necessary. In a second step, the spacer is removed and the membrane that had formed around the spacer is filled with bone graft.

In his current situation in life the patient preferred definite solution with low risk of short- and mid-term-complications and fast return to his everyday-life and family. The patient presented a demanding profession and heavily built physique. In view of the significantly impaired biology with detachment of the soft tissue envelope, impaired arterial perfusion, gross contamination with high risk of infection we therefore indicated talectomy. Long-term results after similar injuries document a high risk of secondary arthritis and repeated interventions, which we hoped to avoid using the described approach [4].

Primary tibiocalcaneal arthrodesis has shown shortening of the injured leg ranging from 1.5 to 4.0 cm [10,11]. So far, only a few cases of tibiocalcaneal arthrodesis have been reported and sufficient data for a comprehensive evaluation is lacking. Alternative graft options for arthrodesis also include the use of vascularised medial femoral head autograft or iliac crest autograft together with arthrodesis [12].

Recently Ruatti reported of a 51-year-old patient with total loss of the talus after a climbing accident [13]. The patient was treated with a custom-made total talar prosthesis. At 2-year follow up results were quite satisfactory as the patient was able to return to his sportive activity.

To our knowledge, this is the first description of of the Masquelet technique as treatment approach for a traumatic loss of the talus. After bony fusion, it allowed the patient to return to full weight bearing with a leg length discrepancy of only 1 cm and low levels of pain. He was able to resume his work as a carpenter with adapted workload intensifying his previous educational duties.

In conclusion, we believe the Masquelet technique to be a viable option in cases of traumatic loss of the talus.

## Funding

There was no funding received.

## Ethical approval

A waiver from the appropriate ethic.

## Consent

The patient read and approved the article and gave his written consent to its publication.

## Author contribution

Ahmed Nabil Abdulazim Surgery, data collection, interpretation, writing of the article.

Martina Reitmaier data collection, interpretation, writing of the article.

Henrik Eckardt Surgery, Masquelet expertise, data collection, interpretation, writing of the article.

Rik Osinga Surgery, data collection, interpretation, writing of the article.

Franziska Saxer Surgery, Masquelet expertise, data collection, interpretation, writing of the article.

## Registration of research studies

n.a.

## Guarantor

Franziska Saxer.

## Provenance and peer review

Not commissioned, externally peer-reviewed.

## Declaration of Competing Interest

There is no conflict of interest with any of the authors.
